# How Health Promoters Can Assess Capacity Building Processes in Setting-Based Approaches—Development and Testing of a Monitoring Instrument

**DOI:** 10.3390/ijerph17020407

**Published:** 2020-01-08

**Authors:** Alexandra Sauter, Verena Lindacher, Jana Rueter, Janina Curbach, Julika Loss

**Affiliations:** Medical Sociology, Department for Epidemiology and Preventive Medicine, University of Regensburg, 93051 Regensburg, Germany; verena.lindacher@muenchen.de (V.L.); jana.rueter@ukr.de (J.R.); janina.curbach@ukr.de (J.C.); julika.loss@ukr.de (J.L.)

**Keywords:** health promotion, evaluation framework, capacity building, monitoring instrument, stakeholder groups, setting-based approaches

## Abstract

*Background*: Health promoters often use stakeholder groups to jointly plan and implement local interventions. Stakeholder groups should take over responsibility to later run the health promotion program independently. Monitoring this process of capacity building can help health promoters improve the quality of the process. Instruments for the systematic assessment of capacity building among stakeholder groups are scarce. The goal of this study was to develop, and pilot test a generic assessment instrument for setting-based capacity building. *Methods*: We drafted a semi-standardized monitoring instrument to be used in stakeholder groups in various settings. This “EVA-protocol” (short for evaluation protocol) was based on capacity building domains e.g., leadership, resource mobilization. It was pilot implemented in a research network on increasing an active lifestyle in various settings. The respective health promoters documented 78 meetings of 15 different stakeholder groups. We performed feedback interviews and member checking among the facilitating health promoters, asking for comprehensibility, length, usability and perceived benefits of the instrument. *Findings*: Data collected in the “EVA-protocol” helped the facilitating health promoters understand the development of competences and capacities in the stakeholder groups and identify factors that favor or hinder the capacity building process. The instrument was rated as user friendly, but it was remarked that it is best filled out by two persons and reflected upon by those to offer the greatest benefit. Not all projects could afford this procedure due to lack of time/staff resources. *Conclusions*: The drafted instrument can serve as quality management tool for health promoters who facilitate participatory stakeholder groups in different settings and intend to build capacities for sustainable health promotion structures.

## 1. Introduction

Health promotion programs are more likely to be successful and sustainable if stakeholders and other setting members are actively involved in the process of needs assessment, program planning and implementation [1]. In order to assure these processes, experts, e.g., health promotion practitioners, need to build coalitions and networks with setting members, e.g., by implementing participatory formats such as stakeholder groups, and build up community strengths as well as resources [2,3,4]. Participatory approaches help communities or organizations acquire competencies in health-related issues and project management, so they can plan and implement needs-based health-promoting interventions. The cooperation of different local actors can facilitate structural changes through equitable engagement of diverse partners [5]. Building networks as well as strengthening competence and awareness in setting members are core strategies of capacity building [6].

The concept of capacity building has become a central element in the theory and practice of health promotion since the World Health Organization (WHO) published the Jakarta declaration in 1997 [7]. The aim of capacity building is to build health structures and health services, to empower organizations and communities to solve their own problems and, in terms of sustainability, to anchor health promotion programs in settings to prolong and multiply health effects [8,9]. Capacity building is meant to make the involved settings responsible for, and more capable of, conducting and maintaining health promotion programs [9]. Capacity building can take place on different levels: individual level (e.g., training coaches), group level (e.g., improving the structure, functioning and learning environment of a project group), organizational level (e.g., integrating competences and skills in processes of schools or sports clubs), and the broader system level (e.g., developing multi-sectoral partnerships between different stakeholders and organizations in a community or rural region) [10,11,12]. It focuses on (community and municipal) structural development as a condition for social and organizational change as well as individual behavioral changes of the actors involved, either working with existing groups and organizations, or establishing new groups (e.g., in communities or schools). These groups should be composed of key persons in the setting, i.e., relevant stakeholders from policy and practice as well as citizens. Building the capacities of groups that consist of stakeholders of a broader organisation or system focuses both on the group level (i.e., the level of the group) and on the organisation/system level (i.e., level of school or community, including policies and structures).

With moving the focus of health promotion efforts towards building capacities of key actors and stakeholders of settings, the interest in evaluating the success of capacity building has risen [6,9]. Different authors have proposed an operationalization of capacity building processes. In a review about models used for capacity building in communities, Liberato et al. [13] identified core domains frequently used for assessing community capacity: resource mobilization, partnership/linkages/networking, participatory decision-making and leadership. These core domains were also included in the dimensions suggested by Gibbon et al. [14] and Laverack and Labonte [15], respectively, and by Goodman et al. [16]; their domains additionally encompass problem assessment, critical reflectiveness/awareness, and relationship to project team. Other authors also focus on learning processes and development of specific skills as part of capacity building, e.g., Hawe et al. [12]. Some authors described indicators for pre-quantified scales in order to locate change in capacity over time.

The capacity building concept has been implemented in numerous health promotion programs worldwide, such as the REACH initiative in Texas - a diabetes prevention project targeting Hispanics [17], the Massachusetts Working on Wellness (WoW) program on workplace health promotion initiatives [18], the Alberta Heart Project, a Canadian initiative to promote heart health in rural communities, workplaces and schools [19], or a German project improving child and parental health in socially disadvantaged neighborhoods [20].

Systematic reviews have outlined a range of theories, models and frameworks used to support capacity building interventions [6,13,21]. Their focus is primarily on the development of relevant domains rather than on specific measurements or instruments that help assess capacity building as an (intermediate) outcome in itself.

Only a few projects used validated measurement instruments to track changes in capacity building [22]. Evaluations often base on self-assessment [23,24]. Self-assessment, though valued for its participatory component, can be challenging because the participating stakeholders and key actors, who are themselves involved in the proceedings of acquiring capacities, must evaluate not only their own work, but also their own competences.

It can therefore be beneficial to complement pre-post-self-assessments of capacity building with a continuous monitoring of the process by a person (e.g., health promoter) who regularly attends (and maybe facilitates) meetings of stakeholder groups, or other formats of setting co-operations. This close monitoring could also help identify factors that favor or hinder capacity development in different settings, as was shown for empowerment processes within participatory groups of lay people committed to planning interventions for healthy nutrition [25,26].

In summary, capacity building approaches have become more and more important over the last ten to fifteen years, as health promoters and decision makers became increasingly aware of the relevance of participatory, bottom-up interventions, of a sense of ownership among setting members, and of sustainability of health promotion measures. Experiences show that capacity building, however, is not always successful. In those interventions where capacity building plays a key role, measuring and monitoring its process and outcome can be helpful in identifying and barriers to capacity building, and how they can be overcome. There is a lack of instruments allowing health promoters to track the capacity building processes among groups they work with. Health promoters may also feel that measuring capacity building, in addition to standard outcome evaluation, may be an additional bureaucratic burden [27,28].

This study is intended to contribute to evaluation research in health promotion by proposing an instrument for health promoters to monitor capacity building across various settings in complex health promotion interventions. The aim of this study was to develop and test a monitoring instrument which considers existing frameworks for capacity building at the organizational and system level in health promotion, and adapt it to the public health issue of active lifestyles, as physical inactivity can be named as one of the core public health problems of the 21st century [29]. The instrument was tested within a research network for the promotion of physical activity regarding content validity, usability, comprehensibility and applicability for measuring capacity building as intermediate outcome variable in different settings.

## 2. Methods

### 2.1. Research Consortium Capital4Health

Capital4Health is a German research network (FKZ01EL1421A) which focuses on active lifestyles within different settings (kindergardens, schools, workplaces, communities, residential homes) [30]. Five empirical subprojects established participatory group processes aimed at planning and implementing interventions to promote physical activity in the respective setting (Table 1). 

In each subproject, groups consisted of setting members, local stakeholders and (mostly) target group members and were facilitated by the project staff (health promoters); they met regularly, discussed needs, developed potential solutions and collectively worked on implementing solutions and interventions in their setting (“co-operative planning process”) [35]. The facilitating health promoters gave informational input and took over (organizational) tasks when needed. All subprojects approved using capacity building strategies when planning the recruitment for and facilitation of the participatory stakeholder groups. The cross-cutting evaluation team was responsible for the overarching evaluation of al subprojects using the newly developed monitoring instrument for measuring capacity building in all stakeholder groups.

**Figure 1:**
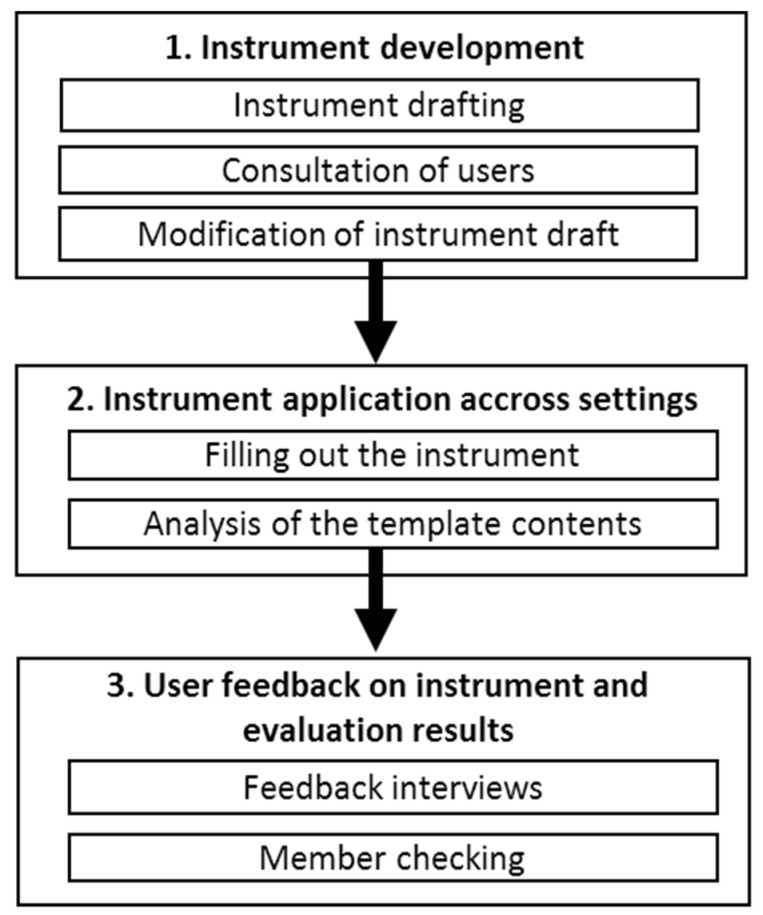
Flow chart of the instrument development process.

A seven-step process was used to develop and test a template for monitoring the capacity building within participatory stakeholder groups, using a qualitative mixed methods design including literature review, semi-standardized interviews and member checking. The development phase took seven months from August 2014 to February 2015. The instrument was applied across the different settings between March 2015 and October 2018 and the user feedback on instrument and evaluation results was generated between January 2019 and June 2019. The individual steps are detailed below (Figure 1). The template was called “EVA-protocol”, which is short for evaluation protocol.

### 2.2. Ethical Considerations

The Ethics Committee of the University of Regensburg granted ethical approval for this study (15-101-0326). The effort and burden for the participants in the study was rated as low. From the point of view of the research team, there was no (ethical) risk associated with participating in the study. All participants agreed to participate in the study and were informed about the objectives and process of the study as well as the processing and publication of the data.

### 2.3. Instrument Development

#### 2.3.1. Instrument Drafting

The draft of the “EVA-protocol” was based on (a) findings from a literature review, (b) a former monitoring instrument used to record the development of empowerment in community groups on healthy nutrition [25,26], and (c) the program logic of the included Capital4Health subprojects, as presented in the respective study designs.

We drafted an instrument using an open answer format, because this seemed to allow us to better understand the processes and dynamics of the interactions and achievements in the various stakeholder groups. We draw on the capacity dimensions identified by Gibbon et al. [14] and Labonte and Laverack [36] as these domains are most frequently found in the literature and fit best in the individual subprojects program logic. Additional, we added further questions on specific skills in physical activity promotion and the establishment of physical activity related infrastructure, as suggested by Hawe et al. [12]. We also included questions on group interaction, as proposed by Curbach et al. [25].

The resulting “EVA-protocol” consists of three parts: (1) questions about the general objectives of each stakeholder meeting and a description of the participants, (2) specific open-ended questions concerning the eight dimensions of capacity building observed in the meeting, (3) questions about specific newly acquired competences of group members. In total, the “EVA-protocol” contains 24 questions. The option “no changes” (as compared to previous meeting) is available for several questions.

#### 2.3.2. Consultation of Users

The protocol draft was presented and discussed on a workshop of the Capital4Health research consortium, and subsequently sent to all project leaders of the five Capital4Health subprojects (1–2 per subproject; in total 6 PIs, 3 co-PIs) as Microsoft Word^®^ document (Microsoft Corporation, Redmond, WA, USA). Thus, every subproject had the opportunity to discuss the draft within its team, with all scientists involved (co-project leaders, PhD-students, senior researchers or research assistants) the aim to establish face validity [37]. All subproject teams were asked to comment on the presented questions in the “EVA-protocol” and the introduction text, and to additionally answer the following questions (in written or oral form):

(1) Is the “EVA-protocol” considered suitable for describing the members of the stakeholder group, their interactions and the development of relevant capacities?
(2) How can the “EVA-protocol” be improved to better fit the program logic, and to improve usability?

#### 2.3.3. Modification of Instrument Draft

Minor revisions were made by all subprojects in oral and written form (via telephone or E-mail), referring to the wording of questions, the need of more examples or clearer explanations. All research teams’ comments were considered by summarizing them thematically, so the respective questions of the “EVA-protocol” were adapted accordingly. All suggestions for improvement and all considered changes were discussed within the evaluation team until consensus was reached. When modifying the questions, it was ensured that, despite specifications, the questions were still generic enough to apply to all five subprojects. The final version of the “EVA-protocol” is detailed in Table 2.

### 2.4. Instrument Application across Settings

#### 2.4.1. Filling out the Instrument

The research teams of all five subprojects were provided the revised instrument for regular use in meetings of participatory stakeholder groups that they facilitated. The format of the “EVA-protocol” was a Microsoft^®^ Word template (Microsoft Corporation, Redmond, WA, USA) to be filled out on the computer. The completed “EVA-Protocol” were sent back to the evaluation team after each meeting via E-Mail. If requested, the members of the evaluation team were also present at the first meetings of the respective stakeholder groups to assist the subproject staff in completing the “EVA-protocol”. After each meeting of the various stakeholder groups, the respective research teams were contacted (via telephone or E-mail) and asked about the meeting and the handling of the template. If necessary, comprehension questions were clarified with the subproject team. The “EVA-protocols” were mostly filled out by one, sometimes two members of the subproject team (in most cases a research assistant or a PhD-candidate). In only a few sub-projects, the contents of the “EVA-protocols” were discussed with the project leader. Table 3 gives an overview of the team-members of the individual subprojects that have regularly filled out the “EVA-protocol”. The “EVA-protocols” were applied until the end of each subproject, i.e., until the point of time when the meetings were discontinued, or when the meeting facilitation was handed over to a local person for the sustained group meetings. The duration of the interventions corresponded to the length of the funding period (3 years) of the subprojects. The evaluation team had no influence on the frequency or number of meetings.”

#### 2.4.2. Analysis of the “EVA-Protocol”

All of the completed “EVA-protocols” were analyzed using deductive content analysis as recommended by Elo and Kyngäs [38]. The completed documents were read into the analysis software ATLAS.ti Version 7 (ATLAS.ti GmbH, Berlin, Germany). Text passages of each document were coded deductively according to the dimensions of the capacity building concept by one author (Alexandra Sauter). To do so, the evaluation team previously developed a codebook with several codes for each capacity building dimension (e.g., dimension “participation”—used codes: equal say, regular participation on meetings, assistance in the implementation of measures). After the end of the first coding—round of A.S., the coded passages were viewed by two other authors (Verena Lindacher., Jana Rueter) and all passages were discussed until consensus was reached [39,40]. Afterwards final codes were brought into a chronological order to highlight the changes of the respective dimensions over time. Results of the singular subprojects were compared to the others in order to identify similarities and differences in the capacity building process across subprojects. This step was discussed with another author (Julika Loss), while constantly returning to the coded protocol passages to check for meaning and context as suggested by Mays and Pope [40].

When analyzing the EVA protocols, attention was also paid on how concordant the answers of the different observants were, and whether they addressed the same dimension of capacity building that was asked in the respective question.

### 2.5. User Feedback on Instrument Use and Evaluation Results

#### 2.5.1. Feedback Interviews

After three years (end of the project phases), we conducted semi-structured telephone—interviews with the research staff of the five subprojects concerning the usability and applicability of the “EVA-protocol”. The following topics were addressed:

(1) How understandable were the questions in the protocol? Did you know what kind of information was expected to be given?
(2) How do you rate the order and layout of the questions?
(3) Was the length of the “EVA-protocol” appropriate? How much time did it take to complete the “EVA-protocol”? Did filling out the protocol compromise your daily project work?
(4) To what extent was the information in the “EVA-protocol” relevant to yourself or to your subproject?
(5) Were there any questions that proved redundant for your subproject or the type of stakeholder group you facilitated?
(6) Are there any further comments or suggestions for improvement?

Feedback interviews were audio recorded and transcribed verbatim. Transcripts were analyzed using inductive content analysis [38]. All text documents were read into the analysis software ATLAS.ti Version 7 (ATLAS.ti GmbH, Berlin, Germany) and coded in an open coding process, to identify relevant text passages. Analytical categories were identified inductively by one author (Alexandra Sauter). After the open coding process, the list of categories was grouped under higher order headings in order to reduce the number of categories, while constantly returning to the coded quotes to check for meaning and context. To ensure rigor of the analytical process, every step was discussed with a second author (Julika Loss).

#### 2.5.2. Member Checking

For trustworthiness of the analyzed data, we used member checking, also known as respondent validation, to ensure accuracy of the analysis and accordance with the subproject teams experiences [39,41]. Analyzed data were presented to the subprojects’ teams via oral presentation and discussed with them. The presentations took place on site in the offices of the individual subprojects.

Aim of the member checking using the “EVA-protocol” results was to check (a) whether the generated data were suitable to give an independent observer a valid impression of the capacity building process within the subproject; and (b) whether the generated data helped the subproject teams gain an understanding of the capacity building process in their specific project setting, which may in turn assist them in planning, improving and evaluating their subproject. For this purpose, final analysis of the completed “EVA-protocols” were reported to the individual subprojects. Questions about the correctness and usefulness of the results were asked to the subproject teams. All comments were documented in written form.

## 3. Results

### 3.1. Instrument Application and Analysis

A total of 78 completed “EVA-protocols” was returned by the five subprojects (91% response rate), covering the 78 meetings of 15 different stakeholder groups over a time span of 31 months. Missing protocols are caused by absence of employees due to illness, or a general shortage of staff and thus a lack of time with an additional meeting protocol.

In 3/5 subprojects, the facilitating health promoters asked the authors of the “EVA protocol” to attend the first (1–3) meetings of their respective stakeholder groups and to assist with filling out the EVA protocol. Protocols completed by the facilitating health promoters were discussed with the evaluation team to clarify any ambiguities. 

The data of all “EVA protocols” were analyzed. Briefly, the analysis has shown that all stakeholder groups were capable of identifying and analyzing assets as well as problems in their settings with regard to physical activity. The groups actively suggested solutions decided upon planning and implementation, and thereby gained authority. 4/5 subprojects implemented certain interventions or activities, e.g., an informal outdoor gymnastics meeting in the community, a modification of playgrounds and play schedules in kindergartens, or new sports offers for employees in setting-based companies. For implementing these activities, the stakeholders have succeeded in establishing links and coalitions with other local partners, thereby securing funding or increasing dissemination, e.g., advertisement of sports events.

“*The pedagogical staff [of the kindergartens] are the stakeholders who are responsible for the implementation of the project actions. They have received support from parents and kindergartens sponsor and have been able to mobilise funds for the purchase of new exercise equipment and for a team training day*”.

(Subproject 01, protocol number 08)

There were also aspects, however, which hampered the capacity building process, e.g., if the composition of the groups was changing over time. The group members were often reluctant to take over the responsibility for specific operational tasks. Sustainable leadership could not be achieved in most stakeholder groups.

“*The meeting was structured in a directive way by the project team, since a decision on the further course of the stakeholder group was required. Possible work packages for the development of new actions as well as the organisation of an information gathering [for community members] were presented to the group. Nevertheless, it was difficult to name stakeholders responsible for single tasks. [A stakeholder explained]: ‘I personally won’t put much work into it [project] anymore*’”.

(Subproject 03, protocol number 08)

By and large, the data that could be retrieved from the “EVA-protocols” covered the dimensions that were expected in the single questions, which hints at a proper validity of the instrument.

### 3.2. Feedback Interviews and Member Checking

#### 3.2.1. Comprehensibility of the Questions

In the users view, the dimension “leadership” was rated to be the most difficult to answer in the “EVA-protocol”, as some facilitating health promoters were not comfortable or familiar with the concept of leadership. Uncertainties related to the characteristics that may or may not constitute “leadership”, or to the numbers of persons that can be considered ‘leaders’ within a stakeholder group. The facilitating health promoters suggested to provide concrete examples for each question to make the assessment easier.

“(...) what indicators do you use to identify whether someone is a leader or not? …If you ask ten people, even experts, you will get different answers. So it would be helpful to spell out the indicators that define a leader [in the “EVA protocol”].”

(IP05)

In those subprojects where the evaluation team members attended one or more stakeholder meetings and filled out the respective “EVA-protocols” themselves, this assistance was appreciated, as those templates served as a guiding example for subsequent “EVA-protocols” to be filled out by the subproject researchers.

#### 3.2.2. Length and Work Load

Overall, the length of the “EVA-protocol” was assessed as reasonable and practicable. The health promoting staff of the subprojects reported that it had taken 15–45 min to complete the “EVA-protocol”, depending on the length and content of the respective group meeting. This time was considered acceptable, as was the length of the “EVA-protocol”.

*“For me, the amount of time necessary to fill out the protocol was all right. Yes, it is additional work, but the scope is okay”*.

(IP01)

#### 3.2.3. Redundancy, Structure and Answer Format

In the feedback interviews the subprojects researchers did not report any question to be unimportant or redundant. It was explained, however, that progress in the stakeholder groups was sometimes slow, so they had difficulties identifying any changes or improvement over time. Consequently, they often chose to note “no change” in the respective fields. Aside from that, the open answer format was rated as useful by all subprojects, as it allowed more space for reflecting the interactions and events in the meetings.

Many respondents suggested to include questions about capacity building strategies that were used by the facilitating health promoters:

*“We don’t have the option to write down which strategies worked or didn’t work [with the group]. We can only present the results. What’s missing here is an open question: ‘What strategies have been employed by the facilitators to change the respective capacities’”*.

(IP02)

#### 3.2.4. Usability

Many of the interviewees pointed out that most of the answers were dependent on the subjective perception of the person filling out the protocol, so the quality of the data could be increased if at least two people discussed the questions and jointly completed the “EVA-protocol”.

“I have filled out the “EVA-protocol” on my own the last few times and have not consulted with X [my colleague]. Which is probably not favorable, because … there’s more subjectivity in it... Last meeting, Y [one specific important stakeholder] wasn’t there, then a certain lack of leadership resulted. And I guess my interpretation is a bit different from X’s [my colleague’s] one. I’m intrigued to learn what X [my colleague] will write down about this topic.”

(IP03)

#### 3.2.5. Benefit for Subproject Teams

3/5 subproject teams explained that using the “EVA-protocol” has been of direct benefit to them during their ongoing project work in terms of process evaluation and quality management. In these subprojects, the “EVA-protocol” served as a basis for reflection of the interactions with and progresses within the stakeholder groups. Thus, it helped identify aspects that could be improved when working with stakeholders, e.g., putting more effort in recruiting new stakeholders who may support the process of implementing physical activity interventions, or the need to better understand the motivations and vested interests of the participating stakeholders. The documentation in the “EVA-protocol” also helped them interpret physical activity outcomes, as it showed how and why capacity building worked out in some institutions and failed in others (e.g., certain schools, certain communities). One interviewee, who joined the subproject at a later stage of the subproject, explained that reading the data in the “EVA-protocol” helped him understand the earlier dynamics and progresses of the stakeholder group meetings in retrospect.

*“[The EVA protocol] would have helped us to answer the question ‘How can we optimize the work in our stakeholder groups’? Is it necessary to address or involve certain people? Is it necessary or relevant to identify a leader? What is it that motivates the people to participate [in the stakeholder groups]? Actually, [we] should have taken a look at the “EVA-protocols” then, because those would have provided good indicators for that”*.

(IP04)

1/5 subprojects filled out the templates, but had not used the data for reflection, process evaluation or quality management. It was not before the member checking, where the analysis of their data was presented by the evaluation team, that the health promoters of this subproject realized that this data would have been meaningful and beneficial to optimize their work with stakeholders, according to their feedback. They stated to regret that they had not discussed and analyzed their data earlier themselves.

“*I can’t remember a great benefit [of filling out the “EVA-protocols”]. But that was also because of a lack of resources (…) one was always pushed to [prepare the next meeting]*”.

(IP04)

One of five subprojects reported that filling out the “EVA-protocol” was of no benefit for the respective subproject. As a reason, the facilitating researchers of this subproject primarily named lack of staff resources to deal intensively with the contents of the “EVA-protocol”.

In addition, two subproject teams considered the protocol so useful for their own project evaluation that they had decided to continue using the protocol in a future project period; one researcher reported that she was now also using the “EVA-protocol” for an intervention working with university stakeholders, independent from the Capital4Health research network.

## 4. Discussion

### 4.1. Principle Findings

In feedback loops with health promoters working in different settings, we could develop a semi-standardized instrument (“EVA-protocol”) that can be used to monitor capacity building processes in stakeholder groups. The instrument is mainly based on eight capacity domains which have been consistently suggested in similar forms by different authors, i.e., participation, leadership, problem assessment, critical awareness, resource mobilization, external linkage, relationship with experts, and project management. These domains were supplemented with questions on specific competencies (here: physical activity) and on group formation aspects.

The data collected in 78 meetings of stakeholder groups in different settings helped understand and compare the dynamics and outcomes of capacity building processes in the different groups over time. Most health promoters who had filled out the “EVA-protocol” considered it a valuable quality management tool for the respective stakeholder group they were working with. They especially appreciated the open-ended format of the questions and the possibilities for discussion and reflection offered by those questions. Some health promoters were struggling with more abstract domains, such as leadership, and pointed out that more concrete examples or indicators would be helpful for utilizing the “EVA-protocol” in a more beneficial way. Inter-subjectivity may also be increased if two health promoters filled out the protocol independently and then compare and discuss their perspectives. The best benefit could therefore be obtained if the health promoters dedicate considerable time and staff resources to collect and interpret the data together; this was also why one subproject did not consider the “EVA-protocol” as helpful, as they could not afford the time to deal intensively with the monitoring instrument or the data collected in it. Giving better guidance on how to rate certain domains, and maybe decreasing the frequency of using the monitoring protocol (from every to every other meeting) may reduce the workload needed for employing the “EVA-protocol” with stakeholder groups, and thus increase its usability.

### 4.2. Strengths and Limitations

There were some limitations in the process of developing and refining the instrument. First, content validity could have been improved further by using an additional expert consensus development approach (e.g., using e-Delphi technique). Second, cognitive interviewing (e.g., think aloud methods) could have been used as an additional form of respondent validation [42]. But as we consulted regularly with the health promoters after they had filled out an “EVA-protocol” of a stakeholder meeting, we obtained continuous feedback on how the monitoring questions were understood and responded to. This information added to the feedback interviews and member checking. Third, the instrument was only tested in health promotion programs focusing on active lifestyles. Further research could examine the applicability of the “EVA-protocol” in other thematic contexts, e.g., nutrition, mental health, or substance abuse.

### 4.3. Comparison with Other Studies

Some studies report the use of instruments that measure capacity building in different settings, with the aim to evaluate a health promotion intervention. There are only few studies, however, that describe the development, pilot testing and/or validation of a capacity building measurement tools. Maclellan-Wright et al. [22] describe the development and establishment of a semi-quantitative instrument to measure community capacity. Their instrument covers nine domains with a total of 26 items and a section for qualitative contextual comments. The development process consisted of six steps including a literature review, a national think tank with experts on measuring community capacity, focus groups on comprehensibility and user-friendliness of the instrument as well as a pilot testing phase. The instrument was to be filled out by community practitioners. Similar to the feedback to our “EVA-protocol”, the instrument was rated as useful to help participants develop project goals, to reflect on the project and to specify further steps. In addition, the authors point out that the instrument could function as an evaluation tool to help identify strength and weaknesses of a project. It also became clear that the qualitative contextual information are important to improve the quality of the data collected. This emphasizes the meaning of open-ended questions in a capacity building protocol.

Nickel et al. [43,44] operationalized a quantitative instrument to measure community capacity, which is based on the work of Laverack and Labonte [15]. Their developed KEQ-instrument (capacity building in small areas/neighbourhoods) consists of 51 standardized items in four domains regarding health and was pilot tested in a long-term prevention program for children and their parents in deprived neighborhoods. The instrument is to be filled out by both professional actors and residents. The primary goal of the KEQ-instrument is to focus on the long-term increase of community capacity, and to identify changes in the outcome over time. The authors reflect that involving residents in the evaluation can lead to a subjective bias in the results of the evaluation. Still, the information obtained through the instrument was considered as helpful for further project work.

### 4.4. Implications for Policy and Practice

Health promoters who intend to build capacities in a certain setting may consider it useful to monitor continuously the capacity development in stakeholder groups or other participatory formats. The pilot tested “EVA-protocol” can serve as a basis for this assessment, and thereby act as a tool for the quality management of a health promotion project. Using the data collected in the protocol, the facilitating health promoters can assess which capacities still need to be developed further, and which (external) resources may still be needed for the progress of the project. Accordingly, changes can be made in the course of the project.

It is also advisable for health promoters to supplement the capacity building data collected in the protocols by a self-assessment of the stakeholders. Other authors suggested that as part of a participatory process, it is important to obtain the stakeholders’ view on the capacities they have developed (e.g., a pre-post evaluation using spider web visualizations [45]). Combining both views—those of the facilitating health promoters and those of the stakeholders themselves—may increase the validity of the evaluation and may also reveal potential misconceptions and divergent expectations.

For health promoters, the evaluation of the outcome of participatory approaches (e.g., environmental changes made in schools or communities, improved health behavior of setting members) may be of primary importance, especially to legitimate the investments made by funders. Monitoring capacity building processes is therefore an add-on which may increase the bureaucratic workload of health promoters. Nevertheless, we would strongly advocate for monitoring capacity building processes in the settings, as they are not only important for the sustainability of health promotion programs but can also help understand why (or why not) changes on the setting or individual level could be achieved. It seems paramount to make the monitoring process as easy and efficient as possible. Therefore, further steps for interactive online versions prompting explanations and allowing access to data from former meetings may be a way to increase the usability of the instrument.

## 5. Conclusions

We drafted and pilot implemented a generic instrument for monitoring setting-based capacity building processes for promoting physical activity in various stakeholder groups. Feedback interviews and member checking with health promoters of five projects pilot testing the instrument confirmed its usability in health promotion practice. Although the application of the semi-standardized tool is resource-intense and should ideally involve two users, the health promoters found it valuable for reflecting upon their work as facilitators of the stakeholder groups and for managing the quality of the setting-based participatory proceedings. Further development of the instrument is needed to minimize the workload of filling it in and to enhance the comprehensibility of some of the more abstract capacity building domains for health promoters.

## Figures and Tables

**Table 1:** Stakeholder group composition in the different subprojects of the Capital4Health consortium.

Setting	Target Group	Aim	Stakeholders Involved in Participatory Groups	Number of Stakeholder Groups	Number of Group Meetings	Name, Source
Child care centers	Children	Increase PA levels of children	Educational staff in child care centers	2	3–8 per group	QueB, [31]
School, university	Pupils	Develop sport-related health competence in pupils at secondary schools	Physical education teachers, pupils, college students, university lecturers	6	3–7 per group	Health.edu, [32]
Workplace	Apprentices	Increase PA of apprentices during workhours	Apprentices and their teachers, company doctor	3	4 per group	PArC-AVE, [33]
Rural communities	Older men	Increase motivation and participation in physical activity programs	Local physicians, members of sport associations, mayor, senior citizens representatives	2	10 per group	ACTION4 Men [34]
Residential homes	Residents at residential homes	Implement a structured physical activity counseling program	Members of the home management, physicians, nurses, social workers, home residents	3	3 per group	PATEN

**Table 2:** Questions for monitoring capacity building in the “EVA-protocol”.

**1. Description of the Group**	
✓ Which tasks and functions do the group members have (e.g., with regard to access to resources/access to target group, specific skills)	
**2. Capacity Building—General Skills**	
Participation	✓ Who participates in the meeting? Which field of work do the participants represent? (e.g., project partner, stakeholder, employee) ✓ Are all participants involved in discussions and suggestions? ✓ How far does the group make decisions on planning and implementing physical activity interventions? Are all participants involved in decisions? ✓ Is the composition of the group suitable for addressing current topic? Why/Why not?
Leadership	✓ Can you identify a leader or leaders in the stakeholder group? ✓ How does leadership show in the interactions of the group? How?
Problem assessment	✓ How far does the stakeholder group identify and analyze problems? ✓ Is the group capable of developing ideas for solutions? In what way?
Critical awareness, asking why	✓ Does the stakeholder group discuss in order to reflect on/question former decisions and actions? Does the group self-analyze its actions and assumptions? How?
Mobilization of resources	✓ Can the group mobilize resources, e.g., funds, material or personnel resources, which are necessary to implement the planned intervention? How are these resources gained?
External linkages, networks, links to others	✓ Can the stakeholder group establish partnerships and coalitions between their group and other actors in the setting? In what way? ✓ Do these links contribute to implementing the activities, e.g., by gaining resources or recruiting new members? How? ✓ Which members are involved in establishing partnerships and networks?
Relationship to facilitating researcher	✓ What are the power relationships between the stakeholder group and the facilitating researchers? Does the stakeholder group assume authority and make its own decisions?
Project management	✓ Do the stakeholder group members have clearly defined roles and responsibilities? ✓ Can the group manage program development and implementation with little or no assistance of the facilitating researchers? How?
**3. Capacity Building—Specific Skills with Regard to Health Promotion/Physical Activity**	
✓ In what way has the group gained expertise and skills with regard to physical activity? ✓ Has the group developed competencies in setting-based health promotion, i.e., changing infrastructure and environment to render physical activity easier?	

**Table 3:** Characteristics of the scientific members of the five subprojects.

Subproject	Setting	Project Team Size	Level of Qualification	Background/Discipline
QueB	Child care centers	4	2 Project leaders	Health science, Social and health science for sports
			1 senior researcher	
			1 research assistant	
Health.edu	School, university	6	1 Project leader	Sport science, Sports pedagogy, Sports education
			2 co-project leaders	
			1 PhD-student	
			2 research assistants	
PArC-AVE	Workplace	3	1 Project leader	Sports science and sports
			1 senior researcher	
			1 research assistant	
ACTION4 men	Rural communities	4	1 Project leader	Medical Sociology, Sports science
			1 co-project-leader	
			1 senior researcher	
			1 research assistant	
PATEN	Residential homes	3	1 Project leader	Sports medicine, Sports science
			2 research assistants	
